# Soybean (*Glycine max* L.) Lipoxygenase 1 (LOX 1) Is Modulated by Nitric Oxide and Hydrogen Sulfide: An In Vitro Approach

**DOI:** 10.3390/ijms24098001

**Published:** 2023-04-28

**Authors:** Salvador González-Gordo, Javier López-Jaramillo, José M. Palma, Francisco J. Corpas

**Affiliations:** 1 Group of Antioxidants, Free Radicals and Nitric Oxide in Biotechnology, Food and Agriculture, Department of Stress, Development and Signaling in Plants, Estación Experimental del Zaidín, Spanish National Research Council (CSIC), Profesor Albareda 1, 18008 Granada, Spain; salvador.gonzalez@eez.csic.es (S.G.-G.);; 2 Instituto de Biotecnología, Universidad de Granada, 18003 Granada, Spain; fjljara@ugr.es

**Keywords:** hydrogen sulfide, lipoxygenase, nitric oxide, peroxynitrite, post-translational modification, protein modeling, tyrosine nitration

## Abstract

Hydrogen sulfide (H_2_S) and nitric oxide (NO) are two relevant signal molecules that can affect protein function throughout post-translational modifications (PTMs) such as persulfidation, *S*-nitrosation, metal-nitrosylation, and nitration. Lipoxygenases (LOXs) are a group of non-heme iron enzymes involved in a wide range of plant physiological functions including seed germination, plant growth and development, and fruit ripening and senescence. Likewise, LOXs are also involved in the mechanisms of response to diverse environmental stresses. Using purified soybean (*Glycine max* L.) lipoxygenase type 1 (LOX 1) and nitrosocysteine (CysNO) and sodium hydrosulfide (NaHS) as NO and H_2_S donors, respectively, the present study reveals that both compounds negatively affect LOX activity, suggesting that *S*-nitrosation and persulfidation are involved. Mass spectrometric analysis of nitrated soybean LOX 1 using a peroxynitrite (ONOO^−^) donor enabled us to identify that, among the thirty-five tyrosine residues present in this enzyme, only Y214 was exclusively nitrated by ONOO^−^. The nitration of Y214 seems to affect its interaction with W500, a residue involved in the substrate binding site. The analysis of the structure 3PZW demonstrates the existence of several tunnels that directly communicate the surface of the protein with different internal cysteines, thus making feasible their potential persulfidation, especially C429 and C127. On the other hand, the CysNO molecule, which is hydrophilic and bulkier than H_2_S, can somehow be accommodated throughout the tunnel until it reaches C127, thus facilitating its nitrosation. Overall, a large number of potential persulfidation targets and the ease by which H_2_S can reach them through the diffuse tunneling network could be behind their efficient inhibition.

## 1. Introduction

Lipoxygenases (LOXs; EC 1.13.11.12) involve a group of non-heme iron enzymes that catalyze the deoxygenation of polyunsaturated fatty acids (PUFAs) into the corresponding lipid hydroperoxides (LOOHs). These products have numerous roles as growth regulators, antimicrobials and antifungals (aldehydes or divinyl ethers), flavors, and as signal molecules such as jasmonates [1,2,3]. Thus, LOXs are involved in diverse physiological plant functions including seed germination, plant growth and development, and fruit ripening and senescence [1,4,5,6], but they are also involved in the mechanism of response to diverse environmental stresses [7,8].

Nitric oxide (NO) and hydrogen sulfide (H_2_S) are two molecules that are endogenously generated in plant cells [9,10]. These molecules and derived molecules designed as reactive nitrogen and sulfur species (RNS and RSS, respectively) participate directly or indirectly in many physiological processes [11,12,13,14,15], and are the response against a wide range of adverse stressful conditions [16,17]. One of the mechanisms to regulate the function of target proteins is through post-translational modifications (PTMs), mainly nitration, *S*-nitrosation, metal nitrosylation, and persulfidation [9,18,19].

Recently, we have reported the presence of several LOX isozymes in different organs of pepper plants, and it was found that the CaLOX IV isozyme present in fruits was regulated by NO [4]. To obtain a deeper knowledge about how the PTMs mediated by NO and H_2_S modulate the lipoxygenase activity and, accordingly, the physiological processes where it is involved, LOX 1 from soybean (*Glycine max* L.) was used and incubated in in vitro assays in the presence of different NO- and H_2_S-derived molecules. The data show that this LOX is susceptible to being negatively modulated by both RNS and H_2_S donors.

## 2. Results and Discussion

Soybean (*Glycine max* L.) is an important crop worldwide since it is an excellent source of proteins, carbohydrates, essential fatty acids, minerals, and vitamins [20,21]. Soybean seeds contain three lipoxygenases designated LOX 1 to LOX 3 [22,23,24], and they can represent up to 2% of the total seed protein [25]. The LOXs from soybeans are of great importance in the food industry since they mediate the flavor, although the oxidation products from LOX activity have also been associated with the development of undesirable flavors in products containing soybean protein [26,27]. For this reason, genetic approaches have been sought for soybean varieties with low levels of *LOX* gene expression or to attempt to eliminate some of the *LOX* genes [28].

LOX catalyzes the deoxygenation of polyunsaturated fatty acids. The most common substrates of plants are linoleic and linolenic acids (C18:2 and C18:3, respectively), and arachidonic acid (C20:4) in animals [1,29,30,31]. In plants, LOXs are designated as 9- and 13-LOXs, according to the carbon position where the oxygen is inserted in both C18:2 and C18:3, but a plant 13-LOX corresponds to a mammalian 15-LOX. In the case of soybean, LOX 1 is considered to function as a 15-LOX, and it has been widely used as a model enzyme for kinetics and mechanistic analyses since it is easy to obtain [32,33,34,35].

Based on previous studies on LOX activity, it has been described that the interaction of NO with LOX can mediate stomatal closure in Arabidopsis [36]. More recently, the analysis of LOX in different pepper plant organs including fruits showed that LOX activity is modulated by NO [4]. Taking the advantage that the purified soybean LOX 1 is easily available, we decided to use it as a model to analyze via in vitro approaches the potential effect of H_2_S and NO, two molecules with signaling properties that can mediate PTMs of proteins, such as persufidation, *S*-nitrosation, metal nitrosylation, and nitration, affecting its functions.

### 2.1. Soybean LOX 1 Activity Is Downregulated by H_2_S and NO Donors

**Figure 1:**
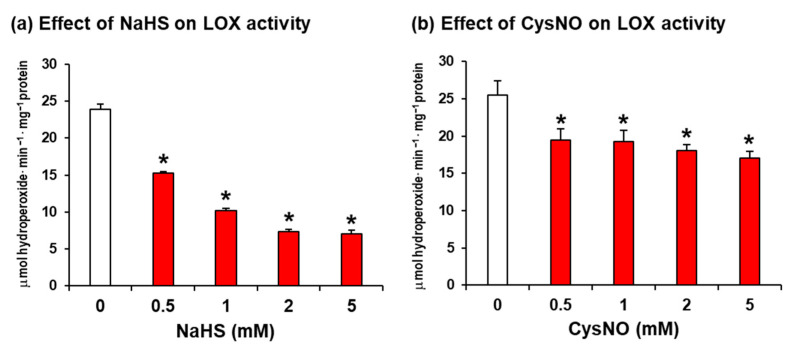
In vitro assays of H_2_S and NO donors on lipoxygenase (LOX) activity from soybean (*Glycine max* L.). (**a**) Sodium hydrogen sulfide (NaHS). (**b**) Nitrosocysteine (CysNO). Treatments with NaHS, as a H_2_S donor, and CysNO, as a NO donor, were conducted by incubating the pure GmLOX with these compounds at 25 °C for 1 h. Data represent the mean ± SEM of at least three different experiments. Asterisks denote significant statistical differences (*p* < 0.05) in comparison with the control (0 mM).

Hence, the soybean LOX 1 was preincubated with increasing concentrations (0–5 mM) of sodium hydrogen sulfide (NaHS) and nitroso-cysteine (CysNO) as the H_2_S and NO donors, respectively, two chemicals that are widely used to study potential PTMs mediated by them [37,38,39]. Figure 1a shows that NaHS exerts an inhibitory effect on LOX activity, from 36% with 0.5 mM NaHS to 71% with 5 mM NaHS. On the other hand, Figure 1b displays that CysNO also exerts an inhibitory effect on LOX activity, ranging between 23% with 0.5% mM and 33% with 5 mM CysNO. These data suggest that this soybean, LOX 1, could undergo processes of persulfidation or *S*-nitrosation/metal-nitrosylation that can trigger its inhibition, with persulfidation being more effective. Early reports using soybean LOX showed that the enzyme activity was reversibly inhibited by NO through the formation of a NO complex of ferrous LOX [40], and later, a similar observation was found in mammalian 15-LOX [41,42]. Our data after the use of CysNO are in good agreement with these previous reports. However, to our knowledge, there are no data about the inhibitory effect triggered by H_2_S.

### 2.2. Soybean LOX 1 Activity Is Also Negatively Modulated by Tyrosine Nitration

**Figure 2:**
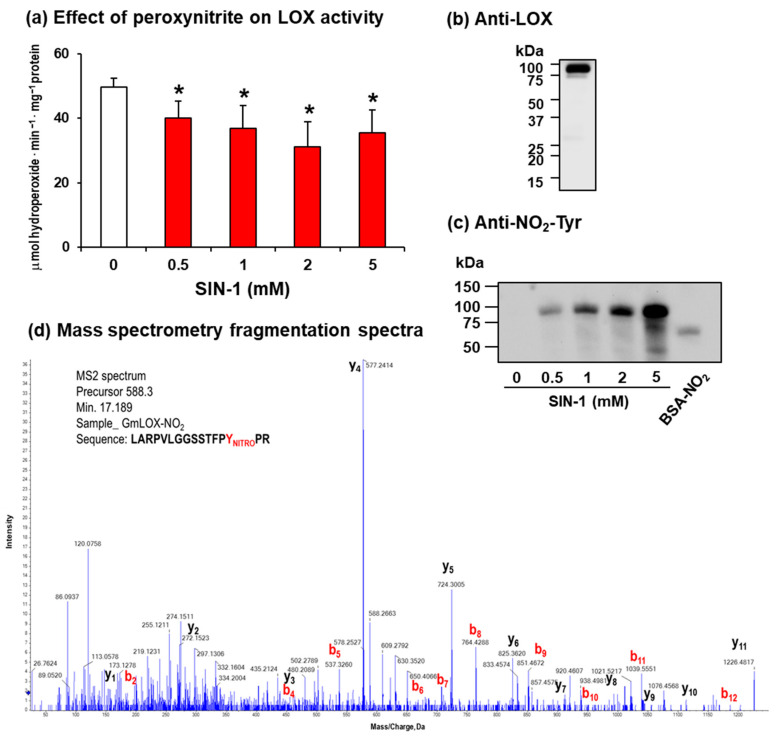
Modulation of lipoxygenase (LOX) from soybean (*Glycine max* L.) using peroxynitrite (ONOO^−^). (**a**) Purified LOX enzyme was incubated in the presence of SIN-1 (0–5 mM) for 1 h at RT, and then the activity was measured spectrophotometrically. Data represent the mean ± SEM of at least three independent experiments. Asterisks denote significant statistical differences (*p* < 0.05) in comparison with the control (0 mM). (**b**) SDS-PAGE-Immuno-blot of purified enzyme (~1 µg per lane) probed with an antibody against lipoxygenase (dilution 1:1000). (**c**) SDS-PAGE (4–20%)-Immuno-blot of purified enzyme (~1 µg per lane) probed with an antibody against 3-nitrotyrosine (NO_2_-Tyr) (dilution 1:1000). Commercial nitrated BSA (BSA-NO_2_) was used as a positive control (1 μg). (**d**) Mass spectrometry fragmentation spectra of the soybean LOX corresponding to the labeled (Y-nitro) version of LARPVLGGSSTFP**Y**PR polypeptide containing nitrated Y214. The fragments corresponding to the main fragmentation series “b”, if the charge is retained on the N-terminus (red), and by “y”, if the charge is maintained on the C-terminus (black), are shown. The subscript in both series (b_n_, y_n_) indicates the number of amino acid residues in the considered fragment from either the N-terminus or C-terminus.

Peroxynitrite (ONOO^−^) is a molecule resulting from the chemical reaction of NO with the superoxide radical (O_2_^•−^). This molecule has great relevance, particularly when it is overproduced under stress conditions, since it can mediate protein nitration, a NO-derived PTM characterized by the addition of a nitro group (-NO_2_) on tyrosine and tryptophan residues [43,44,45,46]. To assess the possible effect of ONOO^−^ on the enzyme activity, soybean LOX 1 was pre-incubated with increasing concentrations of 3-morpholinosyndnomine (SIN-1; 0–5 mM), a recognized ONOO^-^ donor. Figure 2a shows that SIN-1 also exerted an inhibitory effect on LOX activity from 20% with 0.5 mM SIN-1 to 29% with 5 mM SIN-1, thus suggesting that the enzyme could undergo a process of nitration.

The immunoblot analysis of the soybean LOX 1 using an antibody against soybean LOX verified that the molecular mass of the LOX 1 subunit is around 94 kDa (Figure 2b), as had previously been reported [47]. To evaluate if the soybean LOX 1 can undergo nitration, the treated protein with SIN-1 was analyzed via SDS-immunoblot using an antibody against nitro-tyrosine (NO_2_-Tyr). Thus, an immunoreactive band around 94 kDa was observed with an increased intensity signal parallel to the SIN-1 concentration (Figure 2c). To corroborate the reliability of the signal, nitrated bovine serum albumin (69 kDa) was used as a positive control.

To our knowledge, there are no previous data in plants showing that LOX can be nitrated. Therefore, to obtain deeper insights into the nitration mechanisms and to identify which Y residue(s) is (are) likely to be the target(s) among the 35 tyrosines present in the soybean LOX, non-nitrated and peroxynitrite-treated soybean LOX were subjected to trypsin digestion under reducing conditions, followed by LC-MS/MS analysis. Among the list of scanned and identified peptides, only one was identified to contain a nitrated tyrosine. Figure 2d shows the nitrated MS2 spectrum of the nitrated fragment, which corresponds to a peptide of 16 residues L_201_ARPVLGGSSTFP**Y**PR_216_ and a mass of 1764 Da (1719 Da plus 45 Da), which is compatible with Y214 nitration.

Although we have previously mentioned that NO can modulate the activity of soybean LOX via the formation of a NO complex of ferrous LOX [40], to our knowledge, this is the first report showing that the soybean LOX could be also modulated via tyrosine nitration. The information about plant LOX harboring these PTMs is very scarce. Thus, in potato (*Solanum tuberosum*), by using a modified biotin switch assay optimized for potato tissues and nano liquid chromatography combined with mass spectrometry, a LOX (CAA64769) was found to be susceptible to undergo *S*-nitrosation [48]. However, the authors did not check how this NO-derived PTM affected the enzyme activity.

### 2.3. Analysis of the Structure of Soybean LOX 1

Protein folding yields topological features such as cavities and channels that, in many cases, play a role as a location for the active site, binding sites for ligands/cofactors, as well as pathways in the flux of biomolecules. In the case of LOXs, channels are of paramount importance since the active site (metal center) is in the interior of the enzyme and the substrate is a long unsaturated fatty acid (UFA) that needs to be accommodated to reach it.

As a first approach to rationalize the inhibitory effect of the PTMs reported in this work, the PDB entry 3PZW was analyzed with Caver Web [49] to identify the channels that allow access to the metal center. Table 1 depicts the results scored by the throughput parameter that accounts for the probability of a tunnel being used as a channel (it ranges from zero to one, where the higher the value the greater the relevance of the channel). The values of the throughput parameter are similar to those published in a previous study on the gas migration channels using a different PDB entry [50]. The inspection of the tunnels with PyMOL (The PyMOL Molecular Graphics System, Version 1.7) shows that they are interconnected, forming a network of channels (Supplementary Materials Video S1). A closer analysis of Table 1 reveals that, although none of the residues that undergo the PTMs participate in the tunnels with the values of the throughput parameter within the top quartile (i.e., higher than 30), both C127 and C492 participate in the tunnels of the other quartiles. Interestingly, C679 is not identified and C357 appears in tunnel #17 with a throughput score of zero. These results support that the diffusion molecules along the tunnels may reach two of the residues prone to undergo PTMs.

**Figure 3:**
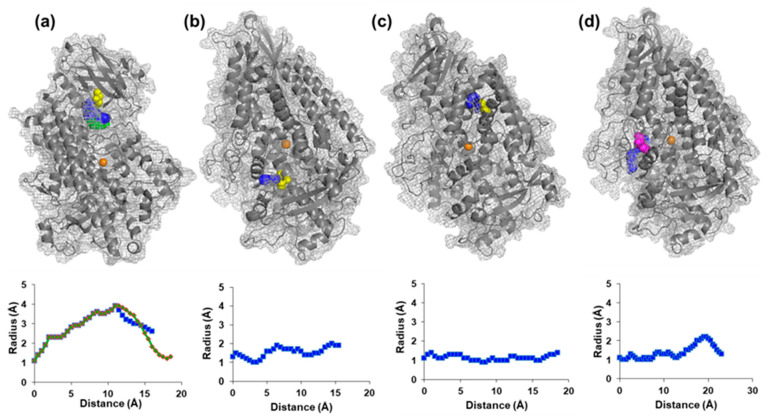
Analysis of the tunnels with throughput scores within the top quartile that connect the surface of the molecule with C127 (**a**), C492 (**b**), C679 (**c**), and Y214 (**d**). Top, location of channels in the structure. Bottom, channel profile. The metal center is shown as an orange sphere, with the cysteine residue in yellow, and Y214 in magenta. Channels are colored blue (#1) and green (#2).

The existence of tunnels that directly communicate the surface with the four cysteine residues and Y492 was evaluated. No tunnel was found for C357, a single tunnel was found for C679, and different possibilities were found for C127, C492, and Y214 (Supplementary Tables S1–S4). For C127, the first quartile comprises two tunnels spanned by a difference in the throughput parameter of 0.13, being 0.77 and 0.64 the outstanding scores for tunnels number one and number two, respectively. For C492 and Y214, a single tunnel is within the first quartile and its difference with the second-best score is 0.25 and 0.14, which is almost the value of tunnel number two for both residues. These results support the feasibility of tunnels number one and number two for Cys127 and number one for C492 and Y214 as channels to reach these residues directly from the surface (Figure 3, top). Residues C357 and C679 seem to be less accessible and, hence, less prone to PTMs.

The treatment with NaHS yields a large inhibition of the enzyme via persulfidation. Thus, the incubation with 0.5 mM NaHS results in the reduction in the activity to 36% and 71% at 5 mM NaHS. Since NaHS is a small molecule, it may reach C429 and C127 either by diffusion through the network of channels that connect the surface of the enzyme with the metal center, or through those that directly communicate both residues with the surface. CysNO, with a larger size, provokes a limited inhibition of the activity, with 23% with 0.5 mM and 33% with 5 mM CysNO. Cysteine residues have been studied in the context of the folding of soybean LOX, and it has been reported that (i) none of the cysteine residues are accessible to 5,5-Dithiobis (2-nitrobenzoic acid) (DTNB, also known as Ellman’s reagent), (ii) a mild treatment with 0.1% SDS exposes one Cys without losing great activity, (iii) the environment of C127 is more hydrophilic than that of C492, (iv) C127 is located at the flexible N-end, and (v) C127 has been proposed as the first cysteine to be exposed [51,52]). In this context, we hypothesized that the small size of NaHS yields the modification of different cysteine residues, including C127, whereas the larger and hydrophilic CysNO only targets C127, provoking a limited inhibition.

Our hypothesis implies that, besides the hydrophilic environment of C127, the channels to C127 have to be wide enough to accommodate the larger CysNO molecule. The analysis of the width of the channels in the first quartile as a function of the distance reveals that the diameter of number one and number two that reach C127 are considerably wider than those heading to the other cysteine, supporting the premise of our hypothesis (Figure 3, bottom). It is important to highlight that none of the potential target residues analyzed in this work are close enough to the metal center to envisage a mechanism of inhibition. This difficulty was not unexpected because, although different studies on soybean LOX have revealed that the rate-limiting step for the oxidation of the UFA is a C-H activation (i.e., bond cleavage) via hydrogen tunneling mechanism, the detailed physical understanding remains elusive due to the inability to obtain X-ray structures of the enzyme–substrate complex. However, it has been reported that the motion within the protein scaffold and the collisions with the solvent provoke a protein quake that leads to thermal activation for the active site chemistry [53]. Moreover, the precise alignment between UFA (substrate) and the Fe^3+^-OH (acceptor) is a key factor that is affected by modest changes in protein structure [54]. Thus, the mutation to Ala of bulky hydrophobic residues that comprise the substrate binding pocket yields decreased rates of activity that have been associated with the alteration of the atom motions, disarranging the reactive configurations, and widening the tunneling barrier, even when the mutated residue is distant from the metal center, as the case of I553 [54]. In this context, the PTMs may disrupt the fine-tuning necessary for the hydrogen tunneling mechanism. In addition, the nitration of Y214 may also affect its interaction with W500, a residue that has been demonstrated to play a role in the substrate binding site and whose mutation to Ala causes a loosening up of the substrate portal [54].

## 3. Materials and Methods

### 3.1. Plant Material

Commercially purified soybean (*Glycine max* L.) lipoxygenase type 1 (LOX 1; EC 1.13.11.12) (Merck Life Science S.L.U., Madrid, Spain) was used for all assays.

### 3.2. Enzymatic LOX Activity and Protein Assay

LOX activity was spectrophotometrically assayed by recording the increase in absorbance at 234 nm, using linolenic acid (C18:3) as substrate according to Surrey (1964), with some modification. Briefly, the assay was performed at 25 °C in a reaction medium (1 mL) containing 100 mM borate buffer pH 9.0 and 16 µM of linolenic acid (Sigma) prepared in 95% dimethyl sulfoxide (DMSO), and the reaction was initiated by the addition of 10 µL purified soybean LOX (dilution 1:4000). One unit of LOX activity is defined as the amount of enzyme that causes an increase in A_234_ of 0.001 per min at pH 9.0 at 25 °C when linolenic acid is used as a substrate.

Protein concentration was determined using the Bio-Rad protein assay (Hercules, CA), with bovine serum albumin as standard. Band intensity was quantified using ImageJ 1.45 software.

### 3.3. In Vitro Treatment with Hydrogen Sulfide (H_2_S) and Nitric Oxide (NO) Donors, and Peroxynitrite (ONOO^−^)

For in vitro assays, soybean LOX samples were incubated at 25 °C for 1 h in darkness with increasing concentrations of different chemicals including sodium hydrosulfide (NaHS) as a H_2_S donor and *S*-nitrosocysteine (CysNO) as a NO donor [16,37,48]. On the other hand, the treatment with 3-morpholinosydnonimine (SIN-1), a peroxynitrite donor, was performed at 37 °C for 2 h in darkness [38]. In all cases, the treatments were performed and the chemical solutions were freshly prepared before use.

### 3.4. SDS-PAGE and Immunoblot Analyses

SDS-PAGE was carried out in 4–20% precast polyacrylamide gel using a Mini-Protean electrophoresis cell (Bio-Rad, Hercules, CA, USA). For immunoblot analyses, the proteins were transferred onto 0.22 µm PVDF membranes using a Trans-Blot^®^ Turbo™ Transfer Starter System (Bio-Rad, Hercules, CA, USA). After transfer, the membranes were used for cross-reactivity assays with either a rabbit polyclonal antibody against native lipoxygenase, type 1 purified from *Glycine max* (Agrisera ASO6 128) diluted 1:1000, or a rabbit polyclonal antibody against nitro-tyrosine (Sigma #N 0409) diluted 1:1000. For immunodetection, an affinity-purified goat anti-rabbit IgG horseradish peroxidase conjugate (Bio-Rad Laboratories) and an enhanced chemiluminescence kit (Clarity™ Western ECL Substrate, BioRad) were used. Chemiluminescence was detected using a Molecular Imager^®^ Gel Doc™ XR documentation system. Band intensity was quantified with the aid of Image J 1.45 software.

### 3.5. Identification of Nitrated Tyrosine in Soybean LOX 1 Using Liquid Chromatography with Tandem Mass Spectrometry (LC-MS/MS)

Soybean LOX 1 (5 µg) was digested with trypsin under standard protocol using reduction conditions with dithiothreitol and carbamidomethylation of the reduced cysteines using iodoacetamide. Once the LOX protein was digested with trypsin overnight, the peptides resulting from the digestion were cleaned of salts using Millipore Ziptip C18 tips and re-suspended in buffer A (water plus 0.1% formic acid) for analysis via LC-MS. Twenty percent of the digested sample was analyzed in each case, using a 20 min gradient, and in an Eksigent 1D equipment coupled to a QTOF model 5600 TripleTOF type mass spectrometer (Sciex). The mass spectrometer operated in DDA (Data Dependent Acquisition) mode, and the MS1 and MS2 spectra were used to launch a search using the Mascot search engine against a database composed of 85,000 entries of *Glycine max* proteins, downloaded from UniProtKB.

### 3.6. In Silico Analyses

Molecular evolution studies were carried out at the UET (Universal Evolutionary Trace) [55] using the coordinates of the structure of the LOX 1 from *Glycine max* at 1.4 Å (PDB entry 3PZW) as input. The evolutionary conservation was ranked according to the rho parameter that deviated from one as the variability (i.e., less evolutionary importance) increased [56].

Channel analyses to explore the possible routes to the residues that undergo PTMs were addressed with Caver Web [49]. The input structure was the PDB entry 3PZW and the starting points were the coordinates of C127, C357, C492, C679, and C4 of the phenolic side chain of Y214, as well as the coordinates of the Fe^2+^ ion to visualize the channels that allow access to the active site. Ligands and water molecules were excluded and probe radius, shell radius, and shell depth were set to 0.9, 3.0, and 4.0, respectively. Tunnels were scored by the throughput parameter that ranged from 0 to 1 (the higher the value, the greater the relevance of the channel) and accounted for the cost of a path, reflecting the probability of a tunnel being used as a channel [57]. Results were analyzed with PyMOL (The PyMOL Molecular Graphics System, Version 1.7).

## 4. Conclusions

LOX is present in the seeds of many cereals and legumes, where it participates in diverse pathways including the mobilization of storage lipids, but also in the generation of different compounds with protective functions against pathogens or abiotic stresses such as jasmonate and divinyl ethers [58,59,60,61]. However, during the mobilization of unsaturated fatty acids, LOX can also generate compounds that cause disagreeable flavors and odors, consequently affecting the quality of derived food such as soymilk and tofu [26,62]. The obtained data indicate that H_2_S and NO can inhibit, to a different degree, the soybean LOX activity, and to our knowledge this is the first report showing that a LOX of plant origin is negatively modulated to a different level via persulfidation, *S*-nitrosation, and tyrosine nitration. Whereas Y214 was identified as a target of the nitration, the analysis of the channels that grant access to the cysteine residues suggests that both C127 and C492 can be persulfidated by NaHS, but CysNO can only act on C127. Therefore, these molecules should be considered new potential tools in the agri-food industry to assess food deterioration as a consequence of LOX activity. Thus, our data could open new windows of research to explain how exogenous H_2_S and NO can modulate LOX activity in horticultural products during postharvest storage whose quality could be affected by the undesirable effects of an increase in LOX activity [63].

## Figures and Tables

**Table 1:** The main features of the tunnels identified by Caver Web connect the metal center with the surface of soybean LOX 1 (PDB entry 3PZW). Channels are scored by the throughput parameter that ranges from zero to one and accounts for the probability of a tunnel being used as a channel.

#	Throughput	Length (Å)	Curvature ^1^	Bottleneck (Å) ^2^	Comments
#1	0.39	25.8	1.5	1.0	
#2	0.33	25.9	1.3	1.1	
#3	0.33	21.6	1.3	0.9	
#4	0.27	30.2	1.5	1.0	C492 (at bottleneck)
#5	0.26	41.3	2.0	1.0	C127
#6	0.22	46.3	2.1	1.0	C127
#7	0.16	41.2	1.5	1.0	C492
#8	0.17	60.6	1.9	1.0	C492
#9	0.16	42.3	1.7	1.0	C127
#10	0.13	60.6	1.7	0.9	C127
#11	0.12	41.5	1.8	0.9	
#12	0.09	47.8	1.5	1.0	C492 (at bottleneck)
#13	0.09	49.4	1.5	1.0	C492
#14	0.07	75.0	2.6	0.9	C127
#15	0.05	68.8	3.3	0.9	C127
#16	0.01	88.3	4.8	0.9	C492
#17	0.00	97.8	3.4	0.9	C357C492

^1^ Ratio between the length of the tunnel and the shortest possible distance between the starting point of the tunnel and the ending point. ^2^ Maximal probe size radius which can fit in the narrowest part of the tunnel.

## Data Availability

Not applicable.

## Supplementary Materials

The supporting information can be downloaded at: https://www.mdpi.com/article/10.3390/ijms24098001/s1.
