# Supplementary material for: Soybean (Glycine max L.) Lipoxygenase 1 (LOX 1) Is Modulated by Nitric Oxide and Hydrogen Sulfide: An In Vitro Approach
Source: Int J Mol Sci. 2023 Apr 28;24(9):8001. doi: 10.3390/ijms24098001 (PMC10178856; doi:10.3390/ijms24098001)
Supplement: Supplementary file 1 [file ijms-24-08001-s001.zip › Table S1 to S4.pdf]

Table S1. Main features of the tunnels identified by Caver that connect C127 with the surface of soybean LOX 1 (PDB entry 3PZW). Tunnels are scored by the throughput parameter that ranges from 0 to 1 and accounts for the probability of a tunnel to be used as a channel.

Tunnel	Bottleneck (Å)	Length (Å)	Curvature[1]	Throughput	Quartile
#1	1.1	15.5	1.6	0.77	Q1
#2	1.1	18.0	1.5	0.64	Q1
#3	1.1	18.4	1.3	0.57	Q2
#4	0.9	16.4	1.8	0.55	Q2
#5	1.1	23.3	1.6	0.55	Q2
#6	0.9	16.6	1.6	0.51	Q2
#7	0.9	16.6	1.4	0.48	Q2
#8	0.9	20.2	1.3	0.45	Q2
#9	1.0	40.4	2.1	0.28	Q3
#10	0.9	49.8	2.5	0.20	Q3
#11	0.9	50.2	2.4	0.19	Q3
#12	0.9	43.4	1.8	0.15	Q4
#13	1.0	72.4	1.8	0.09	Q4
#14	0.9	68.0	1.6	0.09	Q4
#15	1.0	76.7	1.8	0.07	Q4
#16	1.0	90.1	1.7	0.05	Q4
#17	1.0	94.7	1.6	0.03	Q4
#18	1.0	98.3	2.0	0.02	Q4

[1] Ratio between the length of the tunnel and the shortest possible distance between the starting point of the tunnel and the ending point

Table S2. Main features of the tunnels identified by Caver that connect C492 with the surface of soybean LOX 1 (PDB entry 3PZW). Tunnels are scored by the throughput parameter that ranges from 0 to 1 and accounts for the probability of a tunnel to be used as a channel.

Tunnel	Bottleneck (Å)	Length (Å)	Curvature[1]	Throughput	Quartile
#1	1.0	15.0	1.3	0.54	Q1
#2	1.0	25.3	2.0	0.29	Q1
#3	1.0	34.8	1.7	0.24	Q2
#4	1.0	38.8	1.3	0.21	Q2
#5	1.0	37.5	1.3	0.20	Q2
#6	0.9	33.0	2.6	0.19	Q2
#7	1.0	32.9	1.5	0.19	Q2
#8	1.0	54.9	2.5	0.11	Q2
#9	1.0	74.3	1.9	0.09	Q3
#10	0.9	53.7	2.4	0.07	Q3
#11	1.0	63.3	2.2	0.05	Q3
#12	1.0	84.4	1.7	0.05	Q4
#13	0.9	88.7	2.5	0.04	Q4
#14	0.9	82.4	3.1	0.03	Q4
#15	0.9	101.4	6.3	0.01	Q4
#16	0.9	111.3	3.1	0.00	Q4

[1] Ratio between the length of the tunnel and the shortest possible distance between the starting point of the tunnel and the ending point

Table S3. Main features of the tunnels identified by Caver that connect C679 with the surface of soybean LOX 1 (PDB entry 3PZW). Tunnels are scored by the throughput parameter that ranges from 0 to 1 and accounts for the probability of a tunnel to be used as a channel.

Tunnel	Bottleneck (Å)	Length (Å)	Curvature[1]	Throughput	Quartile
#1	0.9	17.7	1.6	0.31	-

[1] Ratio between the length of the tunnel and the shortest possible distance between the starting point of the tunnel and the ending point

Table S4. Main features of the tunnels identified by Caver that connect Y214 with the surface of soybean LOX 1 (PDB entry 3PZW). Tunnels are scored by the throughput parameter that ranges from 0 to 1 and accounts for the probability of a tunnel to be used as a channel.

Tunnel	Bottleneck (Å)	Length (Å)	Curvature[1]	Throughput	Quartile
#1	1.0	22.0	1.6	0.30	Q1
#2	1.0	30.3	1.4	0.16	Q2
#3	0.9	31.3	2.5	0.15	Q2
#4	1.0	46.4	1.9	0.13	Q3
#5	0.9	40.7	1.8	0.12	Q3
#6	0.9	44.9	2.5	0.08	Q3
#7	1.0	62.6	1.19	0.07	Q4
#8	0.9	79.2	2.2	0.06	Q4
#9	0.9	51.2	1.5	0.05	Q4
#10	1.0	92.1	2.3	0.03	Q4
#11	0.9	96.4	4.1	0.02	Q4
#12	0.9	109.0	8.3	0.00	Q4
#13	0.9	119.0	3.5	0.00	Q4

[1] Ratio between the length of the tunnel and the shortest possible distance between the starting point of the tunnel and the ending point
